# Estrogen Receptor-Dependent Regulation of Dendritic Cell Development and Function

**DOI:** 10.3389/fimmu.2017.00108

**Published:** 2017-02-10

**Authors:** Sophie Laffont, Cyril Seillet, Jean-Charles Guéry

**Affiliations:** ^1^Centre de Physiopathologie de Toulouse Purpan (CPTP), Université de Toulouse, INSERM, CNRS, UPS, Toulouse, France; ^2^Division of Molecular Immunology, The Walter and Eliza Hall Institute of Medical Research, Melbourne, VIC, Australia; ^3^Department of Medical Biology, University of Melbourne, Melbourne, VIC, Australia

**Keywords:** sex differences, dendritic cells, estrogens, estrogen receptors, toll-like receptors

## Abstract

Autoimmunity, infectious diseases and cancer affect women and men differently. Because they tend to develop more vigorous adaptive immune responses than men, women are less susceptible to some infectious diseases but also at higher risk of autoimmunity. The regulation of immune responses by sex-dependent factors probably involves several non-redundant mechanisms. A privileged area of study, however, concerns the role of sex steroid hormones in the biology of innate immune cells, especially dendritic cells (DCs). In recent years, our understanding of the lineage origin of DC populations has expanded, and the lineage-committing transcription factors shaping peripheral DC subsets have been identified. Both progenitor cells and mature DC subsets express estrogen receptors (ERs), which are ligand-dependent transcription factors. This suggests that estrogens may contribute to the reported sex differences in immunity by regulating DC biology. Here, we review the recent literature and highlight evidence that estrogen-dependent activation of ERα regulates the development or the functional responses of particular DC subsets. The *in vitro* model of GM-CSF-induced DC differentiation shows that CD11c^+^ CD11b^int^ Ly6c^neg^ cells depend on ERα activation by estrogen for their development, and for the acquisition of competence to activate naive CD4^+^ T lymphocytes and mount a robust pro-inflammatory cytokine response to CD40 stimulation. In this model, estrogen signaling in conjunction with GM-CSF is necessary to promote early interferon regulatory factor (*Irf*)*-4* expression in macrophage-DC progenitors and their subsequent differentiation into IRF-4^hi^ CD11c^+^ CD11b^int^ Ly6c^neg^ cells, closely related to the cDC2 subset. The Flt3L-induced model of DC differentiation in turn shows that ERα signaling promotes the development of conventional DC (cDC) and plasmacytoid DC (pDC) with higher capability of pro-inflammatory cytokine production in response to TLR stimulation. Likewise, cell-intrinsic ER signaling positively regulates the TLR-driven production of type I interferons (IFNs) in mouse pDCs *in vivo*. This effect of estrogens likely contributes to the greater proficiency of women’s pDCs than men’s as regards the production of type I IFNs elicited by TLR7 ligands. In summary, evidence is emerging in support of the notion that estrogen signaling regulates important aspects of cDC and pDC development and/or effector functions, in both mice and humans.

## Introduction

The pro-inflammatory role of estrogens, particularly through their actions on the innate immune system, has been proposed to contribute to the higher immune responses that develop in women (1, 2). Estrogen mediates their effects through two receptors, estrogen receptors (ERs) ERα and ERβ, which are members of the nuclear receptor super family (3). ERα and ERβ function in the nucleus as transcription factors in a ligand-dependent manner. Many studies have shown that mature cells both from the innate and the adaptive immune system express ERs, particularly ERα in mouse (4–10) suggesting that estrogens could regulate their effector functions. Estrogens could also directly act, through their receptors, on progenitor cells to influence the development of myeloid and lymphoid cells (11–17).

Dendritic cells (DCs) are professional antigen-presenting cells that bridge innate and adaptive immunity. They are essential for the activation of naive T cells specific to self or non-self protein antigens and for their subsequent differentiation into effector T (Teff) cells through the secretion of specific cytokines (18). Ontogeny and global gene expression studies indicate that DCs form a separate lineage of mononuclear phagocytes or mononuclear-derived cells, notably on the basis of their continual replenishment from common DC precursors that are distinct from the precursors of monocytes and macrophages (19). DCs are subdivided into three main subtypes: conventional DCs (cDCs), monocyte-derived DCs and plasmacytoid DCs (pDCs) (19). While cDCs are specialized in antigen processing and presentation, thereby converting naive T cells into effector CD4^+^ and CD8^+^ Teff cells, blood monocytes can be rapidly mobilized from the circulation and differentiate into monocyte-derived cells (MCs) with distinct functions such as local reactivation of Teff cells, and superior inflammatory and chemokine production relative to cDCs (18). By contrast, pDCs produce massive amounts of type I interferons (IFNs) in response to viral infections (20).

This review surveys the state of the knowledge regarding estrogen-dependent regulation of DC biology and revisits previously published experiments in light of recent advances in the DC field (18, 19, 21). We will focus on key experimental studies supporting a direct regulatory role of sex hormone estrogens vis-à-vis cDCs and pDCs, which hinges on the ligand-dependent activation of their nuclear receptor, ERα (summarized in Table 1).

**Table 1:** **Effect of estrogen receptor (ER)α signaling on dendritic cells (DCs) and monocyte-derived cells in mouse models**.

Cell population	Models	Effect of ERα signaling
GM-CSF-induced conventional DCs (cDCs)	*In vitro* ERα-deficient mice	(a) Estrogen deficiency or ER-blockade with ICI_182,780_ *in vitro* inhibits the development of CD11c^+^ CD11b^int^ Ly6C^−^ Csf-2-induced-DC (12, 22, 23); partial inhibition of DC development with first generation of ERα-mutant mice still expressing a truncated form of ERα lacking AF-1 domain (12) (b) First demonstration of the unique role of ERα, but not ERβ, in the development of Csf-2-induced-DC; promotion of the maturation and pro-inflammatory cytokine production in response to CD40 and TLR9 stimulation (16) (c) E2 acts on macrophage-DC progenitor (MDP) to promote the development and the effector functions of CD11c^+^MHCII^+^ cells through the upregulation of interferon regulatory factor (IRF)-4 (15, 17, 24); ERα AF-1 is partially dispensable for Csf-2-induced GM-DC development (24)
FLT3L-induced cDCs	*In vitro* ERα-deficient mice	(a) E2 inhibits the development of plasmacytoid DCs (pDCs) and cDCs in Flt3L-driven BM cultures, through direct action on MDP, functional impact of E2/ERα not evaluated in this study (15) (b) E2/ERα enhances the maturation and pro-inflammatory cytokine production (IL-6, IL-12) in response to dual signaling through CD40 and TLR4 or TLR9 stimulation; normal cDC development in the absence of ERα (24)
FLT3L-induced pDCs	*In vitro* ERα-deficient mice	E2/ERα signaling blunts the development of pDCs *in vitro*, but enhances the expression of maturation markers and their effector functions in response to TLR9 *in vitro*; ERα-AF-1^−/−^ pDCs *in vivo* exhibited reduced production of interferon (IFN)-α in response to TLR7 ligand (24)
pDCs (bone marrow)	*In vivo* CD11c-Cre x Esr1^flox/flox^ and Tie2-Cre × Esr1^flox/flox^ deleter models	(a) Promotion of type I IFNs and pro-inflammatory cytokine production in response to TLR7 and TLR9 ligand (10, 25) (b) Enhanced type I IFN production in female bone marrow pDCs stimulated through TLR7 associated with upregulation of *Irf5*, but not *Irf7* expression (25)
Monocyte/macrophages	*Ex vivo* ERα-deficient mice peritoneal macrophages (PM); testicular macrophages; bone marrow-derived macrophages; wound macrophages	Pro-inflammatory effects: (a) E2 supplementation in Ovx mice increases the LPS-mediated production of pro-inflammatory cytokines [IL-1β, IL-6, intracellular nitric oxide synthase (iNOS)] by PM through inhibition of phosphoinositide 3-kinase activity; no effect on TLR4 expression (7) (b) ERα in testicular macrophages promotes CD69, TAM receptor expression, tumor necrosis factor (TNF)-α production macrophage activation, and engulfment of Leidig cells (26) Anti-inflammatory effects, tissue repair: (c) ERα-deficient PM exposed *ex vivo* to LPS or *Mycobacterium avium* exhibited enhanced production of TNF-α (27) (d) ERα promotes alternative macrophage activation and tissue repair in cutaneous wounds healing (28)
Monocyte/macrophages	LysM-Cre × Esr1^flox/flox^ deleter model PM; bone marrow-derived macrophages	(a) Related to Ref. (7), first demonstration that cell-intrinsic ERα-signaling *in vivo* in PM positively regulates the TLR4-dependent production of pro-inflammatory cytokines (IL-1β, IL-6, and iNOS) (8) (b) ERα is necessary for full phagocytic capacity of PM; ERα directly regulates transglutaminase 2 expression; ERα is critical for the maintenance of macrophage metabolism (29)

## ER Expression in Hematopoietic Cells

Estrogen receptors are ligand-inducible transcription factors whose activity is regulated by endogenous estrogens naturally produced over the estrus cycle or during pregnancy. The main endogenous estrogens are estrone (E1), 17β-estradiol (E2), estriol (E3) and estetrol (E4), with E2 as the predominant and most biologically active of the four. In humans, E3 and E4 are selectively produced during pregnancy, by the placenta or by the fetal liver, respectively. ER activities can be modulated also by exogenous ligands, such as synthetic, non-steroidal, non-hormonal agonist, and antagonist ligands known as selective ER modulators (SERMs) (30). SERMs such as tamoxifen and raloxifen are in current clinical use for the treatment of breast cancer and osteoporosis, respectively. It is, therefore, important to understand how such molecules, through ER signaling, may interfere with the development and/or function of immune cells. Despite a growing body of evidence that ER-dependent mechanisms influence both the development and the function of innate immune cells, few studies have sought to determine the relative expression of ER isotypes in different steady-state populations of immune cells. Estrogens exert their effects through two known nuclear receptor family members: ERα, which is encoded by *ESR1* on human chromosome 6, and ERβ, which is encoded by the *ESR2* locus on chromosome 14 (30).

Estrogen receptors are widely expressed in most tissues of both women and men (31). They have also been identified in many different immune cell types including hematopoietic precursors (32). Both ER are expressed in CD34^+^ human hematopoietic progenitor cells (HPCs) found in the bone marrow, but not in HPCs from cord blood (33–36). These findings indicate that hormone-receptor expression is highly regulated in hematopoietic precursors during development. The absence of ER expression in HPCs from cord-blood may protect the fetus from high hormone levels that suppress maternal lymphopoiesis (33) while promoting hematopoietic stem cell (HSC) self-renewal and myelopoiesis (35). Indeed, E2 signaling through ERα was recently reported to promote the cycling of HSCs and multipotent progenitors, thereby supporting the increased demand for erythropoiesis during pregnancy (35).

In mature human cells, monocyte-derived DCs express *ESR1* transcripts and low levels of *ESR2* mRNA (37, 38). *ESR1* expression was comparable between CD4^+^, CD8^+^ T cells, and natural killer cells, which also expressed *ESR2* although at lower levels. B lymphocytes expressed the highest levels of *ESR1* mRNA relative to other leukocytes. Of note, B cells and pDCs displayed the highest levels of *ESR2* mRNA in comparison with any other cell type examined (38). As previously reported (39), expression levels of both ER isotypes were low in blood monocytes, despite evidence in mouse that E2 promoted the TLR4-mediated production of pro-inflammatory cytokines by peritoneal monocyte and macrophage cells, through cell-intrinsic ERα-signaling *in vivo* (7, 8) (Table 1).

In mice, *Esr1*, but not *Esr2*, is expressed in bone marrow progenitor cells, including the common lymphoid progenitor (CLP) and myeloid progenitor (MP) compartments (15). Most of the mature immune cells likewise express *Esr1*, with so far little evidence for *Esr2*-dependent regulation. A notable exception is microglia, the brain-resident macrophages, where TLR-mediated response can be differentially regulated through ERα- or ERβ-dependent mechanisms (40, 41). Murine splenic cDCs and pDCs, as well as bone marrow-derived DCs (BMDCs), also express *Esr1*, but only negligible levels of *Esr2* (32). However, ER expression can be modulated by the tissue microenvironment or by the activation status of the cells, as DCs infiltrating the brain during experimental autoimmune encephalomyelitis (EAE) express ERβ, unlike splenic DCs (42). Moreover, evidence exists that regulatory cytokines, such as IL-27, can induce a dramatic increase in *Esr1* gene expression in cDCs, although the functional consequences of this upregulation are presently unknown (43). ERα appears thus to be the most prominent ER in the human and murine dendritic cell lineages, even though human pDCs express both *ESR1* and *ESR2* transcripts (38).

## Mechanisms of ER Signaling

The ERα and ERβ paralogs are conserved across vertebrate lineages (44). The proteins share five functional and structural domains: two transcriptional activation function domains, AF-1 (A/B domain) and AF-2 (F domain); a DNA-binding domain (DBD; C domain); a hinge domain (D); and a ligand-binding domain (LBD; E/F domain) (45–47).

Following interaction with its estrogen ligand, an ER can regulate cellular function through either of two different mechanisms: the nuclear genomic mechanism, implying direct or indirect binding of the receptor to transcription control regions of targeted genes, and the non-genomic mechanism, initiated by receptors at the cell membrane, which signal through kinase pathways (47).

Occupation of the ligand cavity of the LBD results in conformational changes in the receptor allowing interaction with coactivators, if the ligand is an agonist, or preventing such interactions, if the ligand is an antagonist (48). Transcriptional responses to estrogens were initially recognized to depend on specific interaction of homo/heterodimeric ERs with estrogen-response elements (EREs, GGTCANNNTGACC) found in the promoters of target genes. In addition to this “*classical*” pathway, a “*tethered*” signaling pathway has been described in which ligand-activated ERs can interact with other transcription factor complexes and bind to non-ERE sequences. ERs can thus regulate a number of key transcriptional pathways including those of NF-κB, AP-1, Sp-1, and C/EBPβ (30). Of note, the AP-1 and Sp-1 transcription factor family members are ubiquitously expressed and are known to regulate the expression of several genes important for immune cell development and function (49, 50).

In cancer cells, it has been shown that estrogen-bound ERα can regulate gene expression through its direct or indirect recruitment to target gene promoters, or through its binding to distant regulatory elements or enhancers. ERα molecules can associate with other cofactors such as GATA-3, P2A-g, and FOXA1 to the establishment of chromatin loops, bringing distal ERα-bound elements to the vicinity of the transcription start site in target genes (51, 52). Once bound, ERα associates with coactivator proteins displaying chromatin-modification properties potentiating the recruitment of RNA polymerase II and chromatin remodeling enzymes, such as histone modifying enzymes (HATs, HDACs, and HMTs) (53, 54). It is unknown, however, whether such mechanisms occur in non-reproductive tissues, such as immune cells, in which ER expression is 100-fold lower than in breast cancer cells. Moreover, limited information exists regarding the expression of ER at the protein level in immune cell subsets, as control experiments using appropriate knockout cells are often missing in many experimental settings. However, ER protein levels do not necessarily correlate with mRNA expression, as shown for ER^-^ cancer cells, owing to the coupling of ER cytoplasmic signaling cascades and target gene transcription with rapid ER proteolysis effected by the ubiquitin-proteasome system (55).

Full ligand-dependent ERα activation entails interactions between the AF domains, but the AF-1 and AF-2 transactivation domains can independently activate transcription in a promoter- and cell type-specific manner (46, 56, 57). The relative contribution of AF-1 and AF-2 in ERα activity may depend upon the differentiation stage of the cells (58) or the tissue examined (59). Mice lacking either AF-1 (ERαAF-1^0^) or AF-2 (ERαAF-2^0^) have been recently generated (60–62). Using these animal models, it has been shown that ERαAF-1 was required neither for the E2-dependent vasculoprotective effects (60) nor for osteoporosis prevention (62). AF1 was, however, necessary for breast cancer cell proliferation (63) and uterine growth (60). Unlike AF-1, AF-2 was required for all E2-mediated actions with the exception of the acceleration of endothelial healing (61, 62).

Apart from the “genomic/nuclear” actions that classically rely on AF-1 and/or AF-2 activation, E2 modulates also the activation of several kinases [MAPK, phosphoinositide 3-kinase (PI3K) or PKC], phosphatases, and adenylyl cyclase as well as changes in intracellular calcium (30). These membrane-initiated steroid signaling (MISS) actions are mediated by a pool of intracellular receptors localized to caveolae or rafts at the plasma membrane (64). The palmitoylation of human ERα cysteine 447 appears to be crucial for the targeting of ERα to the plasma membrane through physical interaction with caveolin-1 (65, 66). Recently, a mouse model where ERα Cys_451_, the putative palmitoylation site, was mutated into alanine has provided the first evidence for a tissue-specific physiological role of MISS effects of ERα *in vivo* (67).

These new tools should boost our understanding of the ER-dependent signaling mechanisms by which estrogens regulate the development and function of DC subpopulations *in vivo*. *In vitro* studies using mice mutant for AF-1 and AF-2 domains have already led to advances in this field (24), but we still lack a detailed understanding of the respective contributions of the genomic and MISS effects of ERα signaling to DC biology in physiological settings *in vivo*.

## DC Development and Lineages

Murine DCs are generally categorized into three main groups: cDCs, pDCs and monocyte-derived inflammatory DCs (moDCs). These different populations arise from the common MPs (CMPs) found in the bone marrow (21), which become progressively restricted into macrophage-DC progenitors (MDPs) (68). Although the MDP has been identified as the first progenitor committed to the DC and monocyte lineage, the clonal potential of MDPs to give rise to both monocytes and DCs is still debated (69). Subsequently, a common DC progenitor (CDP), expressing high levels of Flt3 has been identified, which gives rise to both the cDC and pDC lineages (70).

### Conventional DCs

Conventional DCs are subdivided into two main lineages, cDC1 and cDC2 (19). Conventional cDC1s express the chemokine receptor XCR1 (71, 72) and express either CD8α or CD103 (also known as αE integrin), but do not express CD11b. The cDC1 subset is highly efficient at cross-presenting exogenous antigens on MHC class I molecules (73–75) and cDC1s further depend on a common set of transcription factors (Irf-8, Id2, Nfil3, and Batf3) for their development. DCs from the cDC2 subset found in the spleen express CD4 and CD172α and cross-present antigens poorly. They are, however, highly competent at presenting MHC class II-restricted antigens to CD4^+^ T cells. In peripheral tissues, the homologous subset, CD11b^+^ DCs (either CD103^+^ or CD103^−^), do not express CD4 but functionally resemble their splenic counterparts (76, 77). CDC2s mainly depend on Irf-4 (78) and Relb (79) for their development.

### Plasmacytoid DCs

Plasmacytoid DCs are a distinct lineage separated from the cDCs by their transcriptional dependency (particularly on E2-2), their capacity to secrete abundant type 1 IFN, their morphology, and their poor antigen presentation capacity (80–82). They are characterized by their expression of marker molecules B220, Ly49Q, Siglec-H, and Bst2 (CD317, bone marrow stromal cell antigen 2, also known as PDCA-1) (83) in mouse, and BDCA-2 (CD303) and BDCA-4 (CD304) in humans (84).

### Monocyte-derived DCs

Monocyte-derived cells were initially considered an important reservoir for DC development based on the observation that murine and human monocytes can differentiate *in vitro* into “DC-like” cells in the presence of granulocyte-macrophage colony-stimulating factor (GM-CSF, Csf-2) (85, 86). The *in vivo* equivalent of GM-CSF-derived cells has been difficult to identify, and it is now clear that they represent a distinct type of highly plastic cells able to acquire multiple functions in response to environmental cues (87). MCs were initially described as a subset of DCs emerging during inflammation induced by *Listeria monocytogenes* infection and characterized as cells producing large amounts of tumor necrosis factor α (TNF-α) and intracellular nitric oxide synthase (iNOS); as such, they were called Tip-DC (TNF-α and iNOS-producing DCs) (88). MCs were subsequently identified in the response to infection by other pathogens (87, 89) and were shown to differentiate from CCR2^+^ Ly6C^hi^ monocytes recruited to the site of inflammation where they can present antigens to both CD4^+^ and CD8^+^ T cells (89). MCs were shown to be crucial for the induction of Th1 and Th17 immunity in response to pathogens and following immunization in Freund’s complete adjuvant (90, 91). In addition, human MCs have been identified in the joints of rheumatoid arthritis patients and in tumor ascites and preferentially primed Th17 responses (92). Another subset of MCs has been reported to accumulate in inflammatory lymph nodes in response to LPS and to express CD14 and the c-type lectin DC-SIGN (CD209a) (93). However, these cells were subsequently found to express the DC-specific transcription factor Zbtb46 (94). Unlike Tip-DCs, LPS-induced MCs were lost in Zbtb46-DTR deleter mice treated with diphtheria toxin and failed to accumulate in Flt3L^−/−^ mice, suggesting that these inflammatory DCs were more closely related to the cDC lineage (94).

## Critical Role of E2/ERα-Signaling in the Generation of CSF-2-Induced CDCs

Most of the evidence regarding the capacity of estrogens to regulate DC development and function has been obtained using *in vitro* models of DC development, particularly the culture of bone marrow cells in the presence of GM-CSF (Csf-2), a cytokine involved in the development and homeostasis of the myeloid lineage (95). It was initially suggested that the CD11c^+^ MHCII^+^ cells that develop under these conditions were equivalent to the inflammatory DCs arising from monocytes during inflammation *in vivo* (96), as mice lacking Csf-2 or its receptor do not carry any major defects in the development of lymph node and spleen cDCs (21). Csf-2 deficiency has, however, been associated with a reduction in the numbers of CD103^+^ cDCs and CD11b^+^ cDCs in the intestine, dermis, and lung (97). Thus, *in vivo* Csf-2 appears dispensable for lymphoid DC homeostasis, but necessary for the development and maintenance of some tissue-resident DCs, suggesting that Csf-2 is a critical regulator of cDC survival in non-lymphoid tissues (97). Although some studies have suggested that inflammatory or mo-DCs can *in vivo* develop from progenitor cells constitutively lacking Csf-2 receptor (91, 97), evidence also exists for a Csf-2-dependent regulation of mo-DC accumulation in tissues during injury and response to pathogens (98). For instance, Csf-2 neutralization has been recently reported to inhibit the development of a cycling mo-DC population (99). On the other hand, CD4^+^ T cell-derived Csf-2 production can drive the generation of mo-DC during inflammatory diseases (100, 101) and is essential for the pathogenicity of Th17 cells in CNS autoimmunity (102, 103). After subcutaneous immunization with antigen in complete Freund’s adjuvant, Csf-2 has been reported to promote the differentiation of Th1 or Th17 effector cells, by acting on CD103^+^ dermal DCs (104) or mo-DCs (91), respectively. Indeed, Csf-2 is critical for IL-6 and IL-23 production by DCs and for Th17 induction in autoimmune myocarditis (105) and EAE (91). Thus, besides its potential role in promoting the expansion of mo-DCs during inflammation (99–101), Csf-2 appears also critical in assigning the Th17-inducing potential of mo-DCs *in vivo* (91, 105).

Application of this *in vitro* model of Csf-2-induced differentiation to either bulk bone marrow cells or highly purified progenitors, such as MDPs, has shown the presence of E2 in the culture medium to be critical in promoting the development of CD11c^+^ MHCII^+^ cells (106) (Table 1). This was initially demonstrated by using either steroid-free culture medium supplemented with estrogens, pure ER antagonists, or SERMs shown to block ER signaling by estrogens originating from fetal calf serum (FCS) supplementation (12, 15, 16, 22, 107). These studies concluded that estrogens in standard FCS-supplemented medium, probably in the 10^−10^–10^−9^ M range, are sufficient to promote the development of CD11c^+^ DC-like cells. Such estrogen doses are compatible with physiological concentrations in female mice during diestrus and estrus (108). It was then formally established that estrogen-mediated activation of ERα, but not ERβ, was necessary to promote BMDC development in the presence of Csf-2 (16). This was not related to a progenitor deficiency in ERα-deficient mice, as the frequency of MDPs and CDPs in the bone marrow was not affected (24).

**Figure 1:**
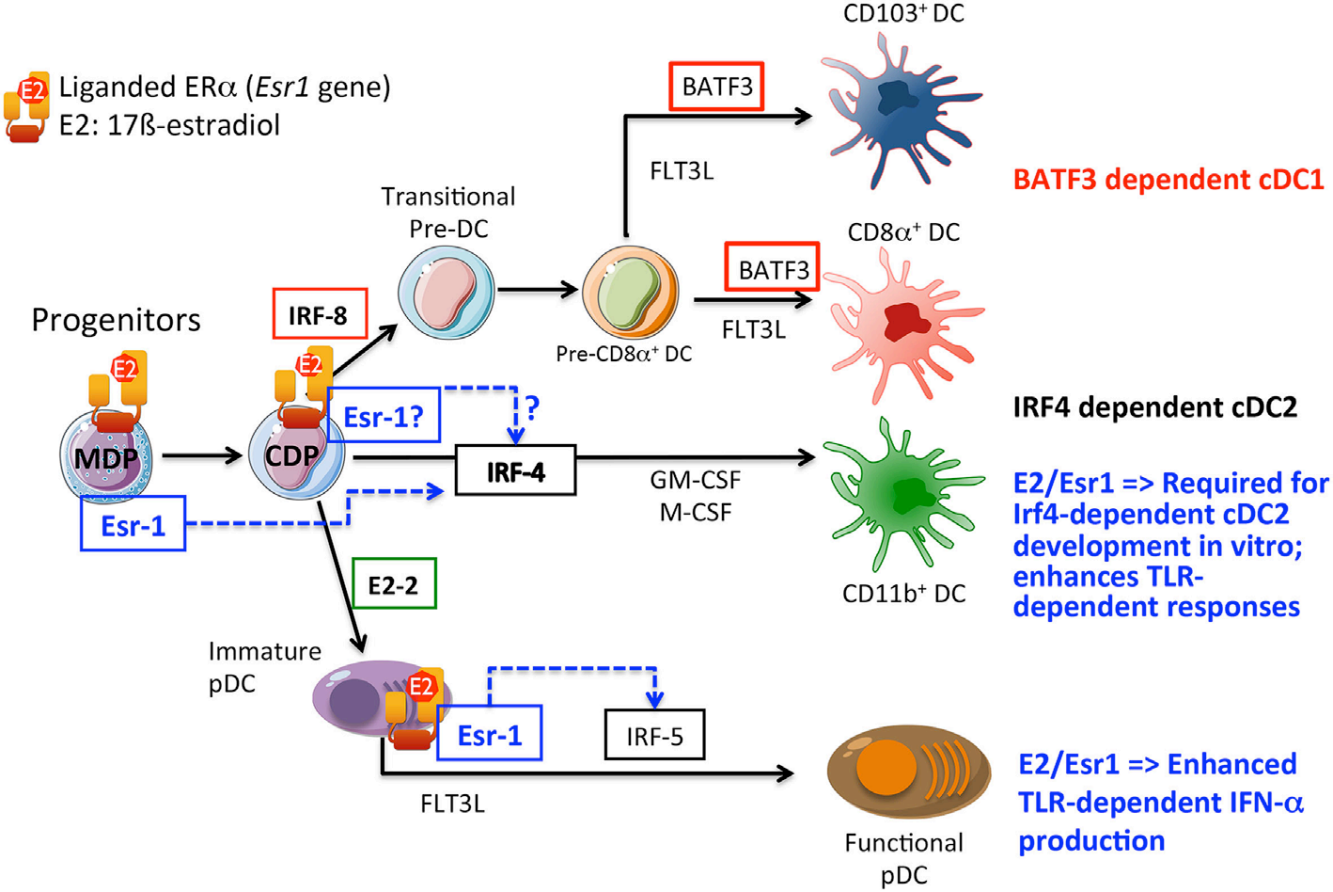
**Growth factors and transcription factors regulating conventional dendritic cell (cDC) and plasmacytoid DC (pDC) development**. A simplified view of the current transcriptional network in DC development is depicted. The plausible developmental stages where E2/ERα-signaling is likely to regulate DC development and/or functions are shown. The common DC progenitors (CDPs) that originate from the common progenitor for monocytes and DCs [macrophage-DC progenitor (MDP)] produce exclusively the cDC and pDC subsets, but have lost the capacity to generate monocytes. Previous works demonstrated that E2 may act directly in MDP to promote interferon regulatory factor (Irf)-4 expression and GM-DC development. Whether E2 may also signal through ERα at later stage in CDP to promote cDC2 development still remains to be established. In the pDC lineage, E2 is likely to signal through ERα to promote optimal TLR-dependent production of type I interferons (IFNs) through the upregulation of signaling intermediate such as Irf-5, and also possibly through additional unknown mechanisms. Solid arrows indicate connections that have been proven, while dotted arrows indicate direct or indirect relationships.

Time-course experiments using the pure ER-antagonist ICI_182,780_ showed that the essential effect of E2 in promoting Csf-2-mediated DC development occurred within the first 48 h of culture (17). This suggested that E2-dependent activation of ERα could regulate the activation state or expression level of transcription factors implicated in DC lineage commitment in the early stages of the differentiation of bone marrow precursors. Indeed, it was subsequently reported that E2 acts directly on highly purified MPs, including MDPs, to regulate GM-CSF-induced differentiation of CD11c^+^ DC-like cells through the upregulation of the transcription factor Irf-4 (15, 17, 24) (Figure 1).

Even though Csf-2 has been widely used to promote the differentiation of mouse and human hematopoietic progenitors and monocytes into cells resembling mouse splenic DCs (85, 109), it was appreciated from the beginning that this culture system harbors heterogeneous cell populations. These include monocyte/macrophage-like cells (85, 109) and CD11c^hi^ CD86^hi^ MHCII^hi^ DCs, which arise spontaneously in the absence of exogenous danger signals (110). A clarification has been provided recently regarding the origin and phenotypic characterization of the cells generated in such *in vitro* model system (111).

Based on their hematopoietic origin and the expression of phenotypic markers, it was reported that the CD11c^+^ MHCII^+^ cells arising in these cultures were comprised of: (i) cMoP-derived macrophages (GM-Macs) defined as CD11b^hi^ MHCII^int^ CD115^+^, and (ii) CDP-derived classical DCs (GM-DCs) expressing high levels of MHCII, PDL-2, and Irf-4 and defined as CD115^−^ CD135^+^ cells. The latter subset was enriched in BMDC cultures generated with GM-CSF and IL-4, and its development was dependent on Irf-4 expression in CD11c^+^ cells (112). Thus, GM-DCs may be more closely related to the CD8α^−^ cDC2 subset, consistent with their expression of CD11b and SIRP-α (111).

These results have important implications for the interpretation of earlier studies on the origin (GM-Macs or GM-DCs) of the estrogen-dependent CD11c^+^ MHCII^+^ cells that preferentially develop in Csf-2-stimulated bone marrow cultures. In previous investigations by different laboratories, the relative expression of CD11b and Ly6C among CD11c^+^ cells was used to better define the CD11c^+^ cell subsets in this model (16, 22), as Ly6C expression appears to represent a reliable marker of inflammatory macrophages, both *in vitro* and *in vivo* (21). From initial studies, it was clear that the CD11b^hi^ Ly6C^+^ population was mostly unchanged by the absence of E2/ERα-signaling. E2, however, preferentially promoted the differentiation of CD11c^+^ CD11b^int^ cells lacking Ly6C expression, likely including the GM-DC population (16, 22). We corroborated this effect in competition experiments using wild-type and ERα-deficient bone marrow cells, which provided direct evidence for a cell-autonomous function of ERα-signaling in promoting the development of CD11c^+^ CD11b^int^ Ly6C^−^ cells. By contrast, the CD11b^hi^ Ly6C^+^ population was reciprocally enriched in estrogen-deprived cultures or in the absence of ERα function in the bone marrow precursors. This cell population may correspond to the “GM-Macs-like” monocyte/macrophage population described by Helft et al. (111). Indeed, we observed a twofold to threefold increase in the occurrence of macrophage-like cells in ERα^−/−^ BMDC cultures (16). This observation correlated with an increased frequency of cells expressing high levels of TLR4-MD2 active complexes and CD11b among ERα^−/−^ CD11c^+^ cells and estrogen-deprived wild-type BMDCs (16, 22). Accordingly, these cells produced massive amounts of IL-6 and IL-12 upon stimulation with LPS, but not in response to TLR9 or CD40 ligands (16, 24). In striking contrast, ERα-signaling promoted the development of CD11b^int^ Ly6C^−^ CD11c^+^ cells capable of producing higher levels of pro-inflammatory cytokines (IL-16 and IL-12) upon TLR9 or CD40 activation than ERα^−/−^ CD11c^+^ cells (16). Moreover, ERα^−/−^ DCs, enriched in GM-Macs, were markedly inferior at stimulating naive CD4^+^ T cells, when compared to BMDCs from E2-stimulated cultures, when given preprocessed antigenic peptides or unprocessed protein antigen (16). Further studies are warranted to explain how E2 promotes the development of GM-DCs in preference to GM-Macs in this culture model. As suggested in Figure 1, E2 could promote GM-DC generation by selectively acting in CDPs and/or by regulating the earlier transition from MDPs to CDPs.

While canceling estrogen signaling in the GM-CSF culture model had no apparent effect on the development of monocyte/macrophage-like cells, this does not necessarily indicate that monocytes or monocyte-derived cells are insensitive to estrogen *in vivo*. In fact, by using the LysM-cre deleter model, we provided compelling evidence that ERα signaling in murine monocytes/macrophages was associated *in vivo* with an enhanced production of pro-inflammatory cytokines by peritoneal macrophages (8). This ERα-dependent effect of E2 on the LPS-driven inflammatory response of macrophages was mediated by inhibition of PI3K activity acting as a negative feedback mechanism of TLR4-induced activation (7, 8). This observation is at odds with other studies indicating that *in vivo*, estrogens exert pro-inflammatory actions on murine macrophages and microglia (40, 41), whereas ERβ ligands are anti-inflammatory (41). This conclusion, however, does not seem to hold for human monocytes/macrophages, in light of reports describing *in vitro* E2-mediated inhibition of LPS-driven production of pro-inflammatory cytokines such as IL-6 (113) through the blockade of NF-κB signaling (114). Whether this effect involves classical ERα is unclear. A recent study showed that the 36-kDa isoform of ERα, which excludes the AF-1 and AF-2 domains owing to alternative mRNA splicing, was strongly expressed in human monocytes and could contribute to the inhibition of NF-κB transcriptional activity through direct interaction in the nucleus (115). Besides macrophages, the effects of estrogen signaling on MCs such as monocyte-derived DCs or TipDCs deserve further investigation. Table 1 summarizes known ERα-mediated modulatory effects of estrogen on the biological responses and functions of DC subsets and monocytes-macrophages in different contexts.

## Irf-4 is a Key Target of ERα Signaling in the Regulation of DC Biology

Interferon regulatory factor-4 is a key transcription factor that plays an essential role in the regulation of Th2 responses through its function in Th2 cells themselves (116, 117) but also in Treg cells (118), M2 macrophages (119) and, as shown more recently, the cDC2 subset (110, 120). Irf-4 was initially shown to regulate the development of CD11b^hi^ CD8α^−^ DCs in the spleen (121), though it was unclear whether Irf-4 was required early during development, or for continued survival or functional competence of Irf-4^+^ DCs in tissues. Recently, DC-specific deletion of Irf-4 has been associated with the absence of CD11b^+^ CD8α^−^ DCs from barrier tissues such as lamina propria, the lung, and the respective draining lymph nodes (122, 123). Mice carrying a DC-specific deficiency for *Irf-4* correctly develop dermal CD11b^+^ CD301b^+^ PDL2^+^ DCs *in vivo*, but these cells fail to localize to the draining lymph nodes (110). This is consistent with the known dependency on *Irf-4* for DC migration from the skin to the draining lymph nodes (124).

Kovats and colleagues identified Irf-4 as a key transcription factor whose expression was strongly up-regulated by E2 in Csf-2/Stat-5-stimulated MDPs (17). Retrovirally delivered *Irf-4* expression in ERα^-/-^ MDPs restored their potential to develop into CD11b^+^ Ly6C^-^ GM-DCs, indicating that high levels of Irf-4 can substitute for the requirement for E2/ERα-signaling during Csf-2-mediated DC differentiation (17). Data from our laboratory, however, point to further ERα-dependent pathways contributing to Csf-2-driven DC development (24). Using mice expressing AF-1- or AF-2 domain-defective ERα, we showed that both AF domains were required for early *Irf-4* expression in Csf-2-stimulated MDPs (24). Intriguingly, in spite of the absence of *Irf-4* upregulation in ERα-AF-1^0^ MDPs, E2 was able to sustain GM-DC development and functional responses to near-equivalent levels relative to wild-type cells at later time points. This observation may explain previous results by others showing substantial E2 effects in GM-DC cultures derived from the first-generation strain of ERα-mutant mice (12), which expressed a truncated form of ERα lacking the AF-1 domain (125), similar to the ERαAF-1^0^ mice used in our study (24). That AF-1 synergizes with AF-2 to enhance Csf-2-dependent expression of Irf-4 in early stage MDPs, but is dispensable at later stages of Irf-4-dependent DC development, suggests indirect mechanisms of Irf-4 regulation by E2-ERα signaling. AF-2 critically controls ERα interactions with most transcription cofactors, but AF-1-specific coactivators have also been described (126). ERα signaling may regulate Irf-4 through the rapid activation of coactivators or corepressors (127), and the induction of other transcription factors whose early expression may require synergistic interactions between both ERα AFs. In the absence of ERα AF1, a longer signaling period could be required for alternative pathways of activation or for the accumulation of rate-limiting factors above the threshold level required to transactivate *Irf-4* expression. This would fit in with the delayed upregulation of Irf-4 observed in ERαAF-1^0^ MP cells as compared to ERα-deficient ones, although many other mechanisms could conceivably be at play (24).

## Relevance of E2/ERα Signaling in CDCs *in vivo*

Little is known regarding the role of ERα-signaling in the homeostasis of cDC subsets in lymphoid and non-lymphoid tissues *in vivo*. Competition experiments in irradiated bone marrow chimeras suggested that newly differentiated splenic CD11c CD11b^+^ cDCs arose from ERα-proficient bone marrow cells in a ~2:1 ratio to cells derived from the ERα^−/−^ donor (17). This model likely represents a pro-inflammatory environment where both GM-CSF and Flt3L may be elevated, suggesting that ERα-signaling *in vivo* may promote the development of CD11b^+^ cDC2 subsets. This conclusion, however, needs further confirmation as few phenotypic and functional markers were used in this study (17).

Experiments by Kovats and colleagues have shown unexpectedly that the skin-derived CD103^+^ cDCs were enriched in the cutaneous lymph nodes of female mice as compared to males (124). Whether this sex bias was due to estrogens was not addressed specifically, but it was independent from Irf-4 expression, in agreement with the currently accepted notion that CD103^+^ cDCs in the skin belong to the Batf-3-dependent cDC1 subset. By contrast, the Irf-4-dependent CD11b^+^ cDC subsets showed no such sex bias (124). Thus, despite strong evidence that E2-ERα-signaling strongly regulates Irf-4-dependent cDC subsets in the *in vitro* Csf-2 differentiation model, it is still unclear how this relates to particular cDC subsets found in lymphoid or non-lymphoid tissues *in vivo*, at the steady state, or during inflammation.

Our search of the ImmGen database at http://www.immgen.org (128) showed that lung CD11b^+^ CD103^neg^ DCs display the highest levels of *Esr1* expression among DC subsets. As mentioned above, several studies have shown that a subset of CD11b^+^ DCs-expressing Irf-4, present in the skin and at the mucosal surfaces in the lung and gut, is crucial in driving CD4^+^ T cell responses and effector T cell development (129). As ERα-signaling controls the level of Irf-4 in Csf-2-stimulated MDPs, and thereby promotes efficient development of the Irf4-dependent CD11b^+^ cDC subset *in vitro*, further investigations are warranted on the impact of cell-intrinsic ERα signaling in cDCs *in vivo* in the context of immune responses against allergens or viruses. A sex bias has in fact been reported for respiratory pathologies such as allergic asthma and influenza virus infection, which are more severe in females than in males (130, 131). It was also reported recently that E2 treatment increased the frequency of Irf-4^+^ DCs in the murine vaginal mucosa, associated with enhanced competence of vaginal DCs to prime Th17 cells *in vitro*. It is unresolved whether this effect of E2 hinged on ERα signaling in DCs (132).

## E2-ERα-Signaling Regulates the Flt3L-Dependent DC Subsets, CDCs and PDCs: *In vitro* Evidence

As mentioned earlier, bone marrow progenitors cultured in the presence of cytokine Flt3L generate the two main conventional DC subsets (cDC1 and cDC2) as well as pDCs. E2 was initially reported to downregulate the development of these Flt3L-driven CD11c^+^ DCs (FL-DCs) through ERα-signaling in MPs (15). Later studies used new strains of ERα mutant mice that allowed the relative contribution of each AF domain of ERα to be examined (24). It was thus shown that this effect of E2 depended on the AF-1 domain, but not on AF-2. Precise analysis of the DC subsets differentiated in the presence of Flt3L confirmed the previous observation that ERα signaling negatively regulates the absolute numbers of FL-DCs *in vitro*. This concerned mainly the development of pDCs, whereas cDC numbers were largely unchanged (24). Importantly, cDCs differentiated in the presence of E2 exhibited an enhanced expression of MHC class II and costimulatory molecules, and an increased capacity to produce IL-12 and IL-6 upon combined stimulation through TLR-4 and CD40. Although E2 likewise decreased the absolute numbers of pDCs, these cells exhibited a more mature phenotype and an enhanced capacity to produce the pro-inflammatory cytokine IL-12 in response to TLR9 stimulation. Similar to cDCs, these effects of E2 on pDC biology were missing in ERα-deficient mice as well as in mice defective for the AF-1 function of ERα (24). This requirement for AF-1 may point to a dominant role for the genomic actions of ERα signaling on target genes, but non-genomic mechanisms could also be at play. Studies in mice lacking the MISS function of ERα will help to resolve this issue.

Thus, in contrast to the Csf2-model of cDC2 development, which is strictly dependent of E2-ERα signaling, the development of Flt3L-driven cDCs and pDCs is not abolished in the absence of estrogens or *Esr1* expression. Instead, our data are consistent with a key role of estrogens in promoting the development of cDCs and pDCs endowed with a more mature functional phenotype, notably characterized by enhanced TLR-mediated responses.

## Functional Impact of E2-ERα Signaling on TLR-Dependent Response of PDCs in Humans

An important issue regards the *in vivo* relevance of the effects of E2 observed *in vitro* on the development and effector functions of cDCs and pDCs, and its translation to human DCs. Addressing the hormonal regulation of immune cells in humans is challenging, and very few studies have as yet been reported. The most remarquable observation probably concerns the human pDC subsets, whose capacity to produce IFN-α in response to TLR ligands has been independently reported to be highly regulated by sex-dependent factors (10, 25, 38, 133, 134). Studying large cohorts of healthy donors, Berghöfer and colleagues demonstrated that pDCs in the peripheral blood of women produced more type I IFNs in response to TLR7 ligands than pDCs from men (133). Subsequent studies showed that this sex bias was in fact due to an increased frequency of pDCs capable to secreting IFN-α among PBMCs from women, rather than a sex bias in IFN-α production on a single cell-basis (10, 134). Support for a key role of estrogens in regulating the TLR-mediated response of female pDCs came from observations that pDCs from postmenopausal women exhibited a reduced TLR7-mediated response by comparison with premenopausal women, which was partially reversed by hormone replacement therapy with E2 (10). Indeed, E2-supplementation of postmenopausal women was found to substantially enhance IFN-α production by blood pDCs not only in response to TLR7 stimulation but also to TLR9 ligands. Importantly, enhanced cytokine production by pDCs was elicited not only by synthetic ligands of TLR7 or TLR9 but also by natural ligands such as self nucleic acid-containing immune complexes present in the sera of systemic lupus erythematosus (SLE) patients (10).

Using an *in vitro* model of Flt3L/IL-7-driven human pDC differentiation from CD34^+^ HPCs, we showed that blockade of ER signaling by the pure ER antagonist ICI_182,780_ in developing human pDCs blunted the IFN-α response to TLR7 ligands (38). Unlike their mouse counterparts, however, human pDCs expressed both ER genes, *ESR1* and *ESR2* (38). It will, therefore, be of importance to investigate the respective contributions of ERα and ERβ to the TLR7-dependent response of human pDCs. The role of either receptor is complex and often mutually antagonistic when expressed in the same cells (135).

## Mechanisms of ERα-Mediated Regulation of Type I IFNs in PDCs

Because they target virtually any cell type in the organism, understanding the mechanisms of immune cell regulation by estrogens is often not straightforward. New mouse models have been generated recently to induce conditional deletions of ER in order to discriminate between direct and indirect *in vivo* effects of estrogens on DC functions (8–10). Using such models, the impact of ER deficiency in specific immune cell types in the context of physiological hormone levels has been assessed (8, 10, 25). These studies pinpointing the cellular targets of estrogens are critical to understanding the molecular mechanisms of ER-regulated immune responses *in vivo*.

Using mice lacking ERα in CD11c-expressing cells specifically, it was established that ERα expression in pDCs was required for the estrogen-mediated increase of IFN-α (10, 25). This *in vivo* cell-intrinsic modulation by estrogen of the TLR7- and TLR9-dependent responses in mouse pDCs (10, 25) was corroborated by evidence from *in vitro* models of murine and human pDC development (24, 38). Altogether, these studies provided new insights into the mechanism of sex bias in the TLR-driven IFN-α production of female pDCs and identified ERα as an attractive target for specific modulation of this pathway.

Interferon-α induction is regulated primarily at the transcriptional level by members of the interferon regulatory factor (Irf) family (136). Human pDCs constitutively express high levels of Irf-5 and Irf-7, and TLR9 engagement in pDCs leads to the activation and phosphorylation of both Irf-5 and Irf-7 (20). Irf-7 is widely recognized as the “master regulator” of type I IFN production (137), while Irf-5 has been shown to be a central mediator of TLR7 signaling in mice (138, 139). In human pDCs, the production of IFN-α through TLR7 or TLR9 stimulation relies on PI3K activities and the phosphorylation of Akt, leading to nuclear translocation of Irf-7 (140). However, a recent study in human pDC leukemic cells challenged the notion of a key regulatory role of Irf-7 in type I IFN production by human pDCs. It was reported that Irf-5, rather than Irf-7, played a non-redundant role in IFN-β and IL-6 production following cell stimulation with TLR9 ligands (36). Thus, while Irf-7 is the master regulator of type I IFN production upon TLR7 or TLR9 stimulation in mouse pDCs, the respective contribution of Irf-7 and Irf-5 to IFN-α induction in human cells is yet to be clarified.

The role of Irf-5 in the differential IFN-α production between females and males was investigated recently (25). Basal levels of Irf-5 were significantly higher in the pDCs from women and positively correlated with the frequency of IFN-α-secreting pDCs upon TLR7 stimulation. Genetic ablation of the *Esr1* gene in the hematopoietic compartment or the DC lineage of female mice reduced Irf-5 mRNA expression in pDCs and IFN-α production upon stimulation with TLR7 ligand-containing vesicles. Of note, Irf-7 expression in bone marrow pDCs was not affected by ERα deficiency (25). These results suggest that estrogens regulate pDC type I IFN production through Irf-5, which may act by enhancing IFN-α production in synergy with Irf-7 (141). This would be particularly relevant to human pDCs, whose capacity to produce IFN-β upon TLR9 engagement has been shown to depend strongly on Irf-5, rather than on Irf-7 (36, 141). However, a direct relationship between E2-ERα signaling and Irf-5 expression has yet to be established. In parallel, it has been reported that estrogen signaling in immune cells upregulated the expression of the endoplasmic reticulum transmembrane protein Unc93b1 (142). This chaperone molecule regulates the trafficking of TLR3, TLR7, and TLR9 from the endoplasmic reticulum to the endosomal compartments, and Unc93b1 knockdown abrogates cytokine production (143). However, estrogen-mediated regulation of Unc93b1 was not assessed in pDCs, and it was unclear whether this regulation involved cell-intrinsic ERα signaling (142). The contribution of Unc93b1 to the sex-bias in the TLR-mediated response of pDCs remains an unsettled question.

While a study has suggested a direct regulation of TLR expression through ERα signaling in human monocyte-derived macrophages (144), we and others have failed to detect sex-dependent modulation of *TLR7* or *TLR9* gene expression in pDCs, at least at the mRNA level in bulk cell populations (38, 133). Confirmation of these results in various TLR7-expressing cells both in mice and human will be necessary, along with analyses of protein expression levels using highly specific antibodies.

Other than TLRs and proteins implicated in their intracellular trafficking, a recent study pointed to ERα-dependent regulation of the cell surface molecule PDC-triggering receptor expressed on myeloid cells (TREM) in pDCs from lupus-prone mice. PDC-TREM is a pDC-specific receptor, a member of the TREM family, which is induced by TLR signaling and mediates IFN-α production (145). ERα deficiency reduced the TLR9-dependent expression of PDC-TREM in pDCs from mice of NZM2410 (lupus-prone) or C57BL/6 genetic background (146). Although PDC-TREM expression is reportedly required for optimal production of type I IFNs by TLR9-stimulated pDCs (145), further studies are needed for any functional links to be established between ERα-dependent regulation of PDC-TREM and changes in the TLR-responsiveness of pDCs.

Despite recent inroads, the mechanistic details of the functional impact of E2-ERα signaling on the TLR-dependent responses of pDCs remain largely unexplored. Apart from the direct regulation of genes implicated in the TLR signaling pathway, ERα could control the expression of key pDC transcription factors, such as E2-2, Spi-B, Irf-8, Irf-7, and Ikaros. One would also expect major effects on the development of pDCs in the bone marrow. This raises exciting questions for investigators, because understanding the mechanisms that promote pDC development and regulate their peripheral function will be key to manipulating this cellular compartment through ER signaling to enforce homeostasis and fight chronic inflammation. The sexual dimorphism in type I IFN production by pDCs could indeed be implicated in the increased susceptibility of women to autoimmunity, and in the sex differences vis-à-vis the acquisition and clinical course of certain viral diseases (1).

## Concluding Remarks

Evidence is emerging that estrogens, through their nuclear receptor ERα, regulate various facets of cDC and pDC develoment and TLR-dependent responses in humans and mice. However, the full demonstration of a direct involvement of this pathway in the sex-biased susceptibility to autoimmunity or infection remains a work in progress. Genetic and cell type-specific *Esr1* deletion strategies have come to the fore as critical tools, and further investigations regarding genes regulated by estrogens in DCs are eagerly awaited. Such unbiased approaches should shed light on the molecular mechanisms set in motion by cell-intrinsic E2-ERα signaling in DCs in the physiological context of the female environment. Another important issue concerns the optimal experimental models to study the role of sex hormones. Although standard estrogen supplementation is useful in addressing certain questions, it does not mirror the estrus cycle and, because of the pleiotropic actions of sexual hormones, may introduce confounding factors, especially in integrated models of autoimmune or infectious diseases. The physiological context of natural estrogen production during the estrus cycle should, therefore, be preferred over experimental models of E2-supplementation, as the only natural instance of sustained steroid hormone production is pregnancy.

The pDC subset appears to be subjected to estrogen-dependent regulation, notably in regard of its functional capacity to produce type I IFNs in response to TLR7 ligands (133, 134). Evidence has arisen that E2-ERα signaling and X-linked genetic factors independently contribute to the strong sex bias in the TLR7-driven response of pDCs (10, 38). Cell type-specific strategies of genetic ablation have recently demonstrated the pivotal role of pDCs in the development of autoantibodies and in the progression of lupus-like syndrome (147, 148). Targeting the ER signaling pathway in pDCs could represent a valuable therapeutic approach to limit the pathogenic production of type I IFN, particularly during the initial stages of SLE. Indeed, early studies suggested beneficial effects of RE antagonists or SERMs in mouse models of lupus (149, 150). Encouraging results were reported from a clinical double-blind trial of fulvestrant (ICI_182,780_) in women with SLE (151). In patients treated for 12 months, an improvement in clinical signs was observed, associated with a reduction of medication dosage (151). Together with our *in vitro* results showing ICI_182,780_ to downregulate the TLR7-type I IFN pathway in developing human pDCs, these clinical outcomes strongly suggest that ER signaling could be amenable to pharmacological manipulation in pDCs. Along this line, we reported earlier that E2 supplementation in postmenopausal women enhances the TLR7- and TLR9-dependent production of type I IFNs (10).

In addition to the well-established sex bias in the incidence and severity of autoimmune diseases, sex differences in the response to viral vaccines have been also clearly documented in humans and in mice (130, 152). Given the central role of DCs in the initiation of adaptive immunity, studying how sex-related factors could regulate the homeostasis and responses of tissue-resident DC subsets at steady state and in the context of vaccination using attenuated viruses or vaccine sub-units is of critical importance. For instance, prophylactic vaccination with HSV-2 glycoprotein D in adjuvant exhibited efficacy in women, but not in men (153). More recently, systems biology approaches have shown that expression of genes associated with TLR and IFN pathways immediately after vaccination with yellow fever virus 17D predicted subsequent adaptive immune responses (154, 155). Interestingly, re-analysis of these data according to the sex of the subjects demonstrated that most of the TLR-associated genes that activate the IFN pathway were upregulated to a greater extent in women as compared to men during the first 10 days after vaccination (130). Although this may suggest that disease protection might be greater in women than in men, this also indicates that the enhanced innate immune response to YF17FD vaccine might also underlie the increased incidence of side-effects in women (130). These examples illustrate the importance of understanding how sex-linked factors could contribute to regulate innate immunity, and particularly DC biology. This may help to explain and predict the heterogeneous responses to vaccines in humans according to sex, not only in the context of infectious diseases but also in cancer immunotherapy.

## Author Contributions

SL, CS, and J-CG participated in the conception and the writing of the review.

## Conflict of Interest Statement

The authors declare that the research was conducted in the absence of any commercial or financial relationships that could be construed as a potential conflict of interest.
